# Accurate and highly interpretable prediction of gene expression from histone modifications

**DOI:** 10.1186/s12859-022-04687-x

**Published:** 2022-04-26

**Authors:** Fabrizio Frasca, Matteo Matteucci, Michele Leone, Marco J. Morelli, Marco Masseroli

**Affiliations:** 1 Dipartimento di Elettronica, Informazione e Bioingegneria, Politecnico di Milano, Milan, Italy; 2 Department of Computing, Imperial College London, London, UK; 3 Center for Omics Sciences, San Raffaele Hospital, Milan, Italy

**Keywords:** Gene expression regulation, Histone modifications, Epigenetics, Interpretability

## Abstract

**Background:**

Histone Mark Modifications (HMs) are crucial actors in gene regulation, as they actively remodel chromatin to modulate transcriptional activity: aberrant combinatorial patterns of HMs have been connected with several diseases, including cancer. HMs are, however, reversible modifications: understanding their role in disease would allow the design of ‘epigenetic drugs’ for specific, non-invasive treatments. Standard statistical techniques were not entirely successful in extracting representative features from raw HM signals over gene locations. On the other hand, deep learning approaches allow for effective automatic feature extraction, but at the expense of model interpretation.

**Results:**

Here, we propose ShallowChrome, a novel computational pipeline to model transcriptional regulation via HMs in both an accurate and interpretable way. We attain state-of-the-art results on the binary classification of gene transcriptional states over 56 cell-types from the REMC database, largely outperforming recent deep learning approaches. We interpret our models by extracting insightful gene-specific regulative patterns, and we analyse them for the specific case of the PAX5 gene over three differentiated blood cell lines. Finally, we compare the patterns we obtained with the characteristic emission patterns of ChromHMM, and show that ShallowChrome is able to coherently rank groups of chromatin states w.r.t. their transcriptional activity.

**Conclusions:**

In this work we demonstrate that it is possible to model HM-modulated gene expression regulation in a highly accurate, yet interpretable way. Our feature extraction algorithm leverages on data downstream the identification of enriched regions to retrieve gene-wise, statistically significant and dynamically located features for each HM. These features are highly predictive of gene transcriptional state, and allow for accurate modeling by computationally efficient logistic regression models. These models allow a direct inspection and a rigorous interpretation, helping to formulate quantifiable hypotheses.

## Background

### Introduction

The regulation of gene expression is the main process allowing cells with identical genome to exhibit substantially different phenotypes. The complexity of this process, reflected in the cellular variety, relies upon a combination of mechanisms that start with the regulation of gene expression [1]. Here, a pivotal role is played by chemical modifications targeting the constituents of nucleosomes, i.e., histone proteins, which are strongly correlated with the alterations in the accessibility of the DNA [2]. These histone modifications (HMs) can be associated with the chromatin structure on the regulating site on the genome, to either enable or block the binding of protein complexes required for gene transcription.

In the last years, aberrations in the combinatorial pattern of HMs have been shown to be connected with several diseases, among which cancer [3]. However, unlike DNA sequence mutations, histone modifications are reversible [4, 5], paving the way to targeted therapies, aimed at developing ‘epigenetic drugs’ to treat cancer in a specific and non-invasive manner [6]. Towards this goal, it is paramount to shed light on the mechanisms relating histone modifications to downstream gene regulation; in this direction, several studies measured HMs with high-resolution data, now cheaply and quickly achievable with Next-Generation Sequencing (NGS) technologies [7–9]. Correlating gene-related HM signals with measured expression levels, such studies have not only performed statistical analyses, but have also built computational predictive models. The best results have been obtained by sophisticated deep learning techniques [10, 11], which attempt to include spatial and compositional inductive biases.

With the present work, we mark a step in a different direction w.r.t. recent computational approaches, and show that it is not necessary—and perhaps not desirable—to resort to complex and hardly interpretable deep learning approaches to effectively model epigenetic transcriptional regulation. Our proposed approach, named ‘ShallowChrome’, consists of a fully interpretable and computationally efficient modeling approach built on top of the well-established pre-processing technique of ‘peak calling’ for ChIP-sequencing data; it is able to obtain state-of-the-art performance in the task of binary classification of gene transcriptional states without using any ‘deep’ technique.

By efficiently computing point statistics on processed and de-noised input signals downstream the peak-calling procedure, our method constructs features that summarise the epigenetic histone activity from a set of contiguous input genomic bins; furthermore, the position of such bins is not enforced a priori as in previous approaches, but is dynamically chosen according to the significance that peak calling attributes them. While delivering effective predictive performance, our method additionally benefits from being highly interpretable; contrary to recent deep learning approaches, not only the feature extraction procedure is intuitive and explainable per se, but also the extracted features are significant enough to be effectively used by a simple logistic regression model, whose parameters can be easily interpreted.

The main contributions of this paper can be summarised as follows:

1. We introduce ShallowChrome, a fully interpretable computational model solving the binary classification of gene activity, based on histone modification features, extracted from de-noised epigenetic signals and used as inputs to logistic regression classifiers;
2. We show that ShallowChrome outperforms the state-of-the-art deep learning approaches presented in [10, 11], with dramatic improvements exhibited over the great majority of the analyzed cell-types/tissues (epigenomes);
3. We present a straightforward procedure to directly inspect the fitted linear classification models and to generate explicative patterns to visually identify the relation between gene-specific epigenetic activity, transcriptional expression and parameters learnt by the model;
4. We evaluate the biological relevance of the extracted patterns by both manual inspection and computational comparison with those from the broadly acknowledged ChromHMM model [12].

### Related work

In the past decade, several pieces of work leveraged on the large amount of NGS data to study the relation between gene-related HM signals and measured expression levels. Besides performing statistical analyses, such studies have also built computational predictive models for regression [13–15] and classification [10, 11, 15].

In regression studies, a function is estimated to predict a real-value measure of gene expression, whilst, in classification, the function is learnt to discriminate between the two classes of active and inactive genes, these being derived by setting some activation threshold over the measured value of gene expression. In both cases, inputs are always considered to be (functions of) the HM signals measured in relevant gene-associated genomic locations, usually those surrounding the genes’ Transcription Start and Termination Sites (respectively, TSS and TTS). In the following, we name these locations as *input-fields*. A common choice for input-fields is to mimic ‘promoter elements’ on the genome, e.g., setting them as symmetric windows of length 10k base pairs around genes’ TSSs [10, 11].

Early studies shared the approach of an epigenetic characterization of genes via feature vectors obtained by stacking together a single real-value feature for each of the histone modifications considered. In this perspective, a crucial design problem is choosing, for every HM, the optimal way of collapsing a signal spanning the entire input-fields into a real number. In [13] and [14] the mean values of the signals over the whole input-fields are taken, whereas in [15] and [16] a ‘binning’ approach is instead adopted. In this last case, input-fields are ‘binned’ into consecutive smaller intervals and then every independent model is learnt on *each* separate bin, or the signal from the bin with the highest correlation with the gene expression is taken as input to a single model.

These ‘featurisation’ approaches present some limitations: in the case of *mean* aggregation, a point statistics is unlikely to convey enough information when computed over raw/untransformed measured signals; binning approaches, on their side, are not able to aggregate the signal from multiple adjacent bins and make use of bins that are statically located over all input-fields. This choice can be limiting, as the most predictive information might be concealed in positions that dynamically depend on the specific HM-gene combination.

Leveraging on these early attempts, recent works [10, 11, 17] have tackled the ‘feature design’ issue from a different perspective. Embracing the spirit of ‘deep learning’, the ‘feature extraction’ stage has been directly included in the learning process. Raw signals throughout the *whole* input-fields are fed into models that learn the best-suited input representations to the current prediction task. Sharing inductive biases similar to those commonly adopted in image recognition and natural language processing, models are built by the composition of established building blocks, e.g., convolutional layers and long short-time memory cells, and consistently with architectural patterns common in the deep learning community.

These models have all shown to attain better results, yet their predictive performance is somehow traded with the ease of interpretability, in particular in the case of [10]. Following works [11, 17] have made a significant improvement leveraging on attention mechanisms [18]; however, even though they allow to explain “what” and “where” a model focuses on when making a prediction, it remains difficult to grasp “why” it focuses on specific features and locations of the input signal. In other words, it is not possible to relate inputs with model parameters in a straightforward manner. Last, these methods usually use a large number of parameters, which have to be learnt; thus, they imply a high computational burden and an inherent high variance, which can be mitigated only when a large number of samples is available.

## Methods

### Problem setting and raw data type considered

Before illustrating our ShallowChrome feature extraction and overall model fitting, as well as its interpretation pipeline, we formalise here the problem our approach solves and the related data types considered.

Our aim is to model gene activity in a given condition/cell-type/tissue through related epigenetic histone modifications; thus, based on these epigenetic features, our computational goal is to solve the binary classification problem of discriminating active genes, i.e., those with low to high measured transcriptional activity, from inactive genes, i.e., those with null to (very) low transcription. In particular, we focus on distinct instantiations of such classification problem, with each single instance being specific to a cell line or human tissue.

Gene activity is quantified in terms of related messenger RNA (mRNA) abundance through RNA-sequencing experiments. Such quantifications are expressed as Reads-Per-Kilobase-Million (RPKM) and a hard threshold is typically set to determine the genes’ class: let ${\bar{t}} \in [0.0, +\infty )$ be the class threshold, then gene *g*, with measured mRNA abundance $t_{g}$, is assigned to class $\tau _{g} = \Omega _0$ (‘OFF’) if $t_{g} \le {\bar{t}}$, or to class $\tau _{g} = \Omega _1$ (‘ON’) otherwise. We choose the class threshold to be problem-instance specific; in particular, we select it as the median value of the mRNA abundances observed within the specific cell line or tissue. This approach is in accordance with [15], where it was first proposed, and is adopted in [10, 11], which are considered state-of-the-art baselines. Nonetheless, we also investigate the possibility of a different and somewhat more sensible approach in Supplementary Section S5, Additional file 1.

Histonic activity is assayed by means of Chromatin ImmunoPrecipitation (ChIP)-sequencing experiments. The output from these experiments is a *tag-align* track, i.e., a genome-wide signal conveying, for each position along the genome, the number of locally aligned read tags; thus, it represents the experimentally measured average presence of a specific histone mark in the cell population. The *tag-align* signals for all the accounted histone modifications represent the raw input that can be used for any classification task; these signals, taken at gene-related input-fields, are directly fed in input to the deep learning models in [10, 11, 17]. In accordance to these articles, we consider the same 5 ‘core’ histone modifications defined by [19]; they are reported in Table 1 along with their biological characterisation.

**Table 1:** The 5 histone modifications considered in this study along with their known biological characterisation (Table from [10])

Histone	Associated with	Functional
Modification		category
H3K4me3	Promoter regions	Promoter mark
H3K4me1	Promoter/Enhancer regions	Regulating mark
H3K36me3	Transcribed regions	Structural mark
H3K9me3	Heterochromatin regions	Repressor mark
H3K27me3	Polycomb repression	Repressor mark

### Feature extraction

While the recently-proposed deep learning methods automatically learn the processed input features best suited for the task at hand, our method adopts instead a standard feature-engineering step performed as a pre-processing step prior to model learning. The main concepts behind our approach are detailed below.

First, many well-known processing techniques can be applied on raw *tag-align* signals to significantly enhance the information value they convey around gene promoter regions, and to characterise their epigenetic conditions. Peak calling algorithms are the most suited to this task: they allow the identification (“call”) of genomic locations where aligned tags are significantly enriched, accounting for background noise and correcting for several sequencing artifacts [20]. Second, by generating de-noised and high confidence output signals, peak calling enables the use of scalar operators on the estimated peak enrichment scores to generate discriminating input features for downstream simple classifiers. In particular, the *max* operator is a good candidate, as it selects the most relevant epigenetic events occurring in dynamic positions within genes’ input-fields, conversely to static locations of bin-based approaches. We show in the following sections that our simple and explicit approach is particularly effective in attaining strong classification results across all epigenomes.

**Fig. 1:**
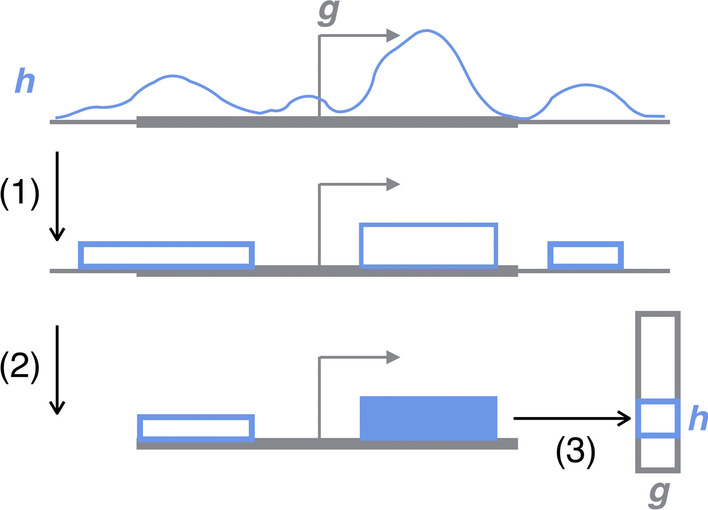
Feature extraction stages for histone mark *h* over gene *g*: ChIP-seq raw and processed signals for *h* are in blue, while the input-field for *g* is depicted in solid, thick grey. (1) *Peak calling:* ChIP-seq raw reads are fed as inputs to a peak calling algorithm to reliably extract read-enriched genomic regions. (2) *Localisation:* Genome-wide peak signals are localised within the defined genes’ input fields. (3) *Extraction:* The max peak enrichment value is extracted as *h*’s value into *g*’s feature vector

Our ShallowChrome’s feature extraction consists of the subsequent steps of *Peak calling*, *Localisation* and *Extraction*; they are detailed below and depicted in Fig. 1.

*Peak calling.* In the first stage of our pipeline, a peak calling algorithm is applied to the ChIP-sequencing signal for each of the considered histone mark modifications. Inputs to the peak calling algorithm are the raw ChIP-seq *tag-align* tracks and, when available, control samples of sonicated chromatin or of non-binding antibody signal. Input controls are valuable to properly eliminate background biases and reliably identify read-enriched genomic regions; nevertheless, several peak calling algorithms provide alternative approaches to cope with the absence of control samples, when they are not available. The output of the peak calling step is a genome-wide track in *bed* format [21] enumerating the called peaks—i.e., those passing a pre-specified significance threshold—along with their genome location, significance and enrichment score.

*Localisation.* The second stage of our pipeline takes as inputs the genome-wide *bed* tracks downstream of the peak calling step and reduces them to the gene-related regions identified by the specified input-fields, which we choose to symmetrically span $l = 10$k base pairs (bp) around each TSS (5k bp upstream, 5k bp downstream), mimicking expanded gene promoters. This choice was performed in accordance to our reference deep learning baselines [10, 11]; it finds justification in the observation that most chromatin modifications regulating gene expression happens in promoters [22–24]. Furthermore, it has been shown in [15] that histonic measurements in promoters are more informative to predict gene expression than those in gene bodies or around the TTSs. In particular, for a gene *g* and a histone mark *h*, the *Localisation* stage outputs a vector ${\mathbf {x}}^{g,h} \in \mathbb {R^{+}}^{l}$ representing a real-valued signal over the whole input-field (*l* base pairs). If a peak has been called over the *g*’s *i*-th input-field position, the ${\mathbf {x}}^{g,h}_{i}$ value is its enrichment (*bed* “signalValue” field); it is set to the null value 0.0 otherwise.

*Extraction.* In the *Extraction* stage, the localised *bed* tracks from all the considered histone mark modifications of the considered condition/cell-type/tissue are jointly taken to compute input feature vectors for all the genes of interest. For a generic gene *g*, its input feature vector is defined to be a vector $\varvec{\phi }^{g} \in \mathbb {R^{+}}^{m}$, where *m* is the number of considered histone mark modifications and the element $\phi ^{g}_{h}$ in the vector summarises the activity of the histone mark *h* in the input-field of gene *g*. We compute $\phi ^{g}_{h}$ as $\phi ^{g}_{h} = \max _{i} x^{g,h}_{i}$, with *i* the *i*-th input-field position of *g*. We choose the non-linear *max* operator because it only retains the most relevant epigenetic activity within a gene input-field in a dynamically localised position. In this sense, our approach builds on top of the ‘best-bin’ idea proposed in [15] that the most predictive information is concealed in localised regions within input fields; however, instead of having a fixed, pre-specified, position, it is dynamically located where the strongest epigenetic signal is measured for a specific gene-HM pair.

### Model fitting and analysis

As resulting from the last stage of the feature extraction procedure, for a given condition/cell-type/tissue each gene *g* is described by an input feature vector ${\varvec{\phi }}^{g}$ along with its binary class $\tau _{g}$. Input feature vectors and target classes can be gathered in the input matrix ${\varvec{\Phi }} \in \mathbb {R^{+}}^{n \times m}$ and target vector ${\mathbf {T}} \in \{0, 1\}^{n}$, for *n* genes and *m* histone marks, to form a supervised learning dataset $D = \langle {\varvec{\Phi }}, {\mathbf {T}} \rangle$, which can be properly split in training, validation and test sets to learn and evaluate a classification model.

In principle, any standard classification algorithm can be learnt on *D*; here, we choose the *logistic regression* classifier. This choice is motivated by the fact that logistic models have small variance, are fast to train, and are inherently interpretable. Even though, in principle, these models suffer from larger bias w.r.t. more complex models, we show in the following that the quality of the extracted input features is sufficient to generally outperform state-of-the-art deep learning models.

The use of logistic regression allows us to interpret the result of model training by jointly relating input signals and model weights. In this kind of models each trainable weight-parameter is specifically tied to a single input feature; thus, it is possible to directly investigate the role and importance of each histone mark in determining class predictions. Therefore, HMs can be related directly to the sign and magnitude of the learnt parameters. Importantly, statistical tests on model weights can be additionally performed to assess whether a particular histone mark has a significant contribution in determining the activation status of genes. These kinds of statistical analyses, especially when compared across epigenomes, are particularly valuable to understanding the fundamental mechanisms by which epigenetic markers act to regulate the activity of genes. Methods to interpret ensemble or neural network models have been recently developed, with remarkable results by approaches based on Shapley additive explanations [25] or paired-input knockoffs strategies [26]. Nonetheless, simpler and more direct interpretation is possible in the case of generalised linear models, for which well studied and developed statistical methods for inference on coefficients are available. At a more specific level, the role and importance of HMs is conveyed by gene-wise patterns constructed by the element-wise product of learnt weights with input feature values. Let $\varvec{\phi }^{g,e}$ be the feature vector for gene *g* and epigenome *e*, and ${\varvec{w}}^{e}$ be the vector of model weights learnt on *e*. We define the *weighted input vector* for *g* on *e* as $\varvec{\psi }^{g,e} = \varvec{\phi }^{g,e} \odot {\varvec{w}}^{e}$ and refer to it, more generally, as *g*’s regulative pattern. Notice that, if $b^{e}$ is the learnt bias parameter for a model, output logits are given by $y^{g,e} = b^{e} + \sum _{i=1}^{m} \varvec{\psi }_{i}^{g,e}$, with *i* indexing the considered histone marks. The weighted input vectors are therefore an effective means to assess the relative importance of single histone marks in determining the predicted response value.

### Implementation

We implemented the described ShallowChrome method in Python programming language. At https://github.com/DEIB-GECO/ShallowChrome the source code is available together with already preprocessed data and Jupyter notebooks to reproduce all paper results. The notebooks also allow for interactive analyses of the models.

## Results

### Binary classification of gene transcriptional states

In this Section we demonstrate the effectiveness of the feature extraction and modeling scheme of ShallowChrome by discussing the results obtained on a series of experiments.

#### Setup

In order to compare ShallowChrome with state-of-the-art approaches, we verbatim followed the experimental setting of [10, 11]. In particular, we instantiated the binary classification problem described above over the same 56 human epigenomes (i.e., cell-types/tissues, see Supplementary Table S1, Additional file 1) and 19,802 protein coding genes, assigning gene class labels based on a median threshold over RPKM gene expression measurements and describing the epigenetic activity via the five core histone marks reported in Table 1. Following [10, 11], the training/validation/test splitting scheme assigns approximately one third of genes to each set, with 6601 genes for training, 6601 for validation and 6600 for test. In order to provide reliable estimates on the performance of our method, for each epigenome we randomly generate 10 of the above splits and compute average test performance along with its standard deviation. Classification performance is measured as the Area Under Receiver Operating Characteristic curve (AUROC). Results are also reported in terms of F1-score and Area Under Precision Recall curve (AUPR) in Supplementary Section S3, Additional file 1.

#### Data

In accordance to [10, 11], all datasets related to histone mark activity and gene expression quantification have been retrieved from the database of Roadmap Epigenomics Mapping Consortium (REMC) [27], a public resource of human epigenomic data produced from hundreds of cell-types. In REMC, the considered HMs have been uniformly profiled across all the epigenomes in this study, and gene expression has been quantified over the whole human genome. Importantly, while we collect the same RPKM abundance measurements as in [10, 11], for ChIP-seq data, instead of retrieving *tag-align* tracks to quantify the activity of the chosen HMs, we start from processed data after peak calling, according to our proposed approach.

REMC provides *bed* files for peaks called on raw alignments with the MACSv2 [20] software for different calling configurations, resulting in the three following output formats: ‘bed NarrowPeak’, ‘bed BroadPeak’ and ‘bed GappedPeak’. While ‘bed NarrowPeak’ processed measurements capture narrow continuous regions of enrichment in histone ChIP-seq signals, the remaining formats allow for broader domains of enrichment, or even subsets of domains in the case of the ‘bed GappedPeak’. Within the scope of the ShallowChrome pipeline, the output peak configuration (whether narrow, broad or gapped) and all other peak calling parameters (such as the Poisson *p*-value thresholds in MACS) are amenable to hyperparameters; their choice is usually specific to each considered input histone mark and epigenome, and can be driven by domain knowledge or simply be the result of model selection procedures. For further details on the peak calling procedure applied in REMC we refer the reader to https://egg2.wustl.edu/roadmap/web_portal/processed_data.html.

Other than HM processed alignments in the form of called peaks, in input to the *Localisation* phase of our feature extraction are the input-fields for the considered genes, which we defined as symmetric windows spanning 10k bp over each TSS. We took the same exact TSS annotations as in [10, 11], which were shared by the authors. The *Localisation* phase requires joining gene input-fields with the bed track for each HM; this was performed via the publicly available GenoMetric Query Language (GMQL) [28, 29] toolkit^1^, which provides access to a high-level, declarative language allowing scalable genomic queries over heterogeneous data formats.

#### Models and hyperparameters

As introduced above, we fit a simple logistic regression model for each of our experiments. The only hyperparameters considered were thus the peak format (‘narrowPeak’, ‘broadPeak’ or ‘gappedPeak’) for the chosen input HMs.

In [10, 11], given a training/validation/test split, the best set of hyperparameters, *H*, is chosen amongst the possible configurations ${\mathcal {H}}$ as the one optimizing validation performance. Here, we follow the same approach, but, as previously mentioned, we independently run the tuning and evaluation procedure over $k = 10$ different randomly generated dataset splits, and report mean and standard deviation statistics over the obtained test AUROCs. Details about ${\mathcal {H}}$ are in Supplementary Section S2, Additional file 1, while Supplemetary Section S3 of the same file reports model performance in terms of F1-score and AUPR.

#### Performance analysis

**Fig. 2:**
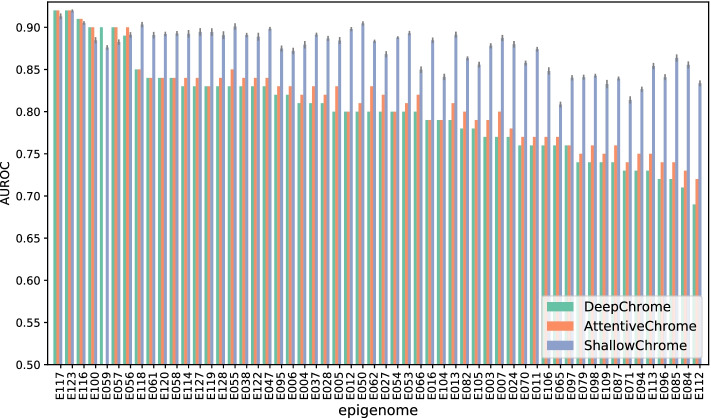
Test AUROC scores for DeepChrome, AttentiveChrome and ShallowChrome. Results are reported on the 56 considered epigenomes, indicated with their respective REMC code (see Supplementary Table S1, Additional file 1, for association between REMC codes and epigenomes). Results for AttentiveChrome have been manually reproduced by the authors of this manuscript. It was not possible to reproduce the result on epigenome E059, thus none about it is reported in the present figure. See Supplementary Section S8, Additional file 1, for further details

ShallowChrome resulted performing, on average, significantly better than the state-of-the-art deep learning models in [10, 11]. Figure 2 illustrates the performance attained by ShallowChrome, AttentiveChrome and DeepChrome [10] for each considered epigenome, while Table 2 reports mean, median, maximum and minimum test performance across all the considered epigenomes. In the following, we will compare the performance of our proposed approach with that of DeepChrome on a per-epigenome basis, and, as for AttentiveChrome, in an aggregated fashion.

ShallowChrome is consistently more accurate than DeepChrome: its AUROC significantly outperforms DeepChrome one on 50 out of 56 classification tasks (i.e., epigenomes), with a performance gap of 14.57% on epigenome *E084* (“Fetal_Intestine_Large” tissue); in the 6 epigenomes where ShallowChrome does not outperform DeepChrome, the difference in performance ranges between only 0.04% and 2.39% (1.15% on average). Interestingly, we notice that, while DeepChrome exhibits a significant performance degradation on certain tissues, with a difference of  24% between the top and the worst scoring epigenomes, our model is consistently more robust, with a mean test AUROC always above 0.8.

**Table 2:** Aggregated statistics on the test results for DeepChrome, AttentiveChrome and ShallowChrome computed across the 56 considered epigenomes

Statistic	DeepChrome	AttentiveChrome	ShallowChrome
Mean	0.8008	0.8133	0.8737
Median	0.8009	0.8143	0.8829
Max	0.9225	0.9218	0.9196
Min	0.6854	0.7237	0.8084

Values reported for AttentiveChrome are those corresponding to the model configuration attaining best result statistic. ShallowChrome statistics are computed over mean test performances for each epigenome

ShallowChrome compares more than favourably also against AttentiveChrome [11]. We observe from Table 2 that our method has the best mean (6.04% gap), median (6.86% gap) and minimum test scores (8.47% gap), and is only slightly behind baselines just for the maximum test performance (0.29% gap). These aggregated results further demonstrate the superiority of our proposed approach, which in addition is consistently more robust and provides much more easily interpretable results than deep learning state-of-the-art approaches.

Additional sensitivity analyses are enclosed in Supplementary Section S3, Additional file 1. We investigate the use of alternative evaluation metrics and study the impact of several design choices, including that of using only *subsets* of input signals, aggregation statistics different than *max*, and a more expressive classifier. The interesting upshot from these analyses is that our feature selection strategy of applying a *max* aggregator over signals downstream of peak calling accounts for most of the predictive accuracy of our pipeline. We iterate that the use of a simple logistic regression model allows to ease intepretation at best, still retaining reasonable and extremely robust performance across epigenomes. We refer readers to Supplementary Sections S3.5, S3.6 in particular for details on this last set of experiments.

Finally, let us remark that the employed data splitting scheme assigns approximately one third of genes to each training, validation and test set; accordingly, the training set contains exactly 6601 samples/genes. This strategy was originally adopted by the authors of the DeepChrome and AttentiveChrome state-of-the-art models and thus guarantees fairness in our result comparison. However, we reckon that this may represent a conservative splitting strategy. If, on one hand, it is valuable to assess the behavior of predictive models in scenarios of higher data scarcity, on the other hand, more complex deep learning models may benefit from larger training datasets. It would be interesting to perform comparative performance analyses for increasing amounts of available training data, and also using validation data, after hyperparameter tuning, for the final model fit before assessing accuracy on test data. We defer these endeavours to the future development of this work.

### Biological interpretation and evaluation

Besides accuracy and robustness, our method has the advantage of being easily interpretable. To this aim, in this section we analyze the regulative HM patterns ShallowChrome generates for a gene that is differentially expressed across three different epigenomes. Additionally, we verify the validity of the extracted patterns by comparing them with chromatin-state characterisations from the widely acknowledged ChromHMM model [12].

#### Gene-wise regulative patterns

The PAired boX 5 (PAX5) is a key gene responsible for the regulation of B-cell commitment. Within the haematopoietic system, it controls the differentiation, function and identity of B lymphocytes [30], and is reported to be exclusively expressed from the pro-B to the mature B cell stage [31]. Similarly to [11], we consider this gene as a test-bed example, and investigate the HM regulative patterns extracted by our model across the epigenomes H1-hESC, GM12878 and K562, where PAX5 is, respectively, in ‘OFF’, ‘ON’ and ‘OFF’ state.

*Weighted input-patterns* for PAX5 were generated according to the procedure described in Section “Model fitting and analysis”; additionally, we conducted statistical fitting tests (*Z*-tests) to assess the significance of the contribution for each of the input HM signals. For each of the three considered epigenomes, we chose a gene split where PAX5 appeared in the test set, and took the ShallowChrome model trained on the corresponding training set. This ensured PAX5 to have no contribution in the estimation of the model weights used to generate its HM pattern. We also augmented the *weighted input patterns* by reporting the model bias: this learnt parameter conveys a prior on the prediction of gene state that is independent on epigenetic regulations and helps in interpreting the absolute contribution of histonic signals in determining transcription activation.

**Fig. 3:**
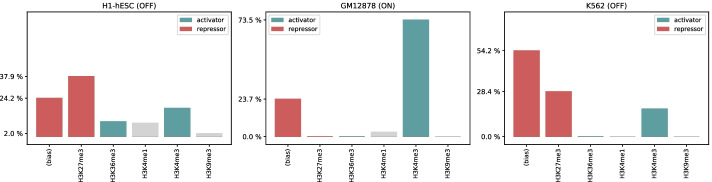
Normalised regulative patterns extracted for gene PAX5 on epigenomes H1-hESC, GM12878 and K562; model bias is included to ease interpretation. Relative contributions to gene activation are in red when negative (repressors), in green when positive (activators) and in grey when found not to be statistically significant (*Z*-test *p*-value $> 0.0001$)

Figure 3 depicts the results of the described interpretation procedure: the PAX5 regulative patterns from ShallowChrome coherently recapitulate the interplay of the considered HMs in determining PAX5 transcriptional activity. In H1-hESC and K562 cell lines, where the gene is not expressed, the contribution of repressor mark *H3K27me3* is prominent. Furthermore, in H1-hESC the presence of marks *H3K36me3* and *H3K4me3* on PAX5 promoter suggests the chromatin to be open and ready to be activated, i.e., the gene to be in a poised (bivalent) state. Transitioning from H1-hESC to the differentiated GM12878, the repressor mark *H3K27me3* does not perform any significant regulation, while a critical role is played by the promoter mark *H3K4me3* that clearly explains the activation of PAX5. We remark that the ‘repressor’ and ‘activator’ roles for *H3K27me3* and *H3K4me3*, respectively, correspond to the sign of the associated weights, which have been autonomously inferred from the data by our model. Interestingly, the model has consistently learnt a negative bias in all the cases: this can be interpreted as an ‘activation threshold’, and captures a tendency for genes to remain in a default inactive state in the cases where no epigenetic activity is measured [32]. Applying ShallowChrome on an appropriate dataset, this analysis can be repeated on any gene of interest, thus dissecting the exact contribution of the different HMs to the expression of the gene in the specific context where the experiment is performed.

#### Matching regulative patterns with chromatin states

To further investigate the validity of ShallowChrome, we compared our extracted gene-wise regulative patterns to chromatin characterisations inferred from the ChromHMM model [12] across all the 56 epigenomes considered.

ChromHMM is a widely adopted [33–35] multivariate hidden Markov model that explicitly models the combinatorial presence or absence of histone marks along the genome. Unsupervisedly trained on multiple epigenomic tracks over many cell lines, it learns to characterise “hidden” chromatin states and the relation among them. In particular, chromatin states are associated with *emission vectors* defining discrete enrichment probability distributions over the input epigenetic markers.

Our validation consists in matching extracted gene-wise regulative patterns against ChromHMM emission vectors, and in studying their correlation with gene activation predictions in output from our model. To this aim, we selected a 15-state ChromHMM model trained on the same core set of HMs considered in our work, which is publicly available in the REMC repository. This model has been learnt on the virtual concatenation of highest-quality consolidated data corresponding to 60 epigenomes. Further details on the model, input data and parameters can be found on the REMC website^2^. As for our model, we retained the same dataset split as in Section “Gene-wise regulative patterns”, and extracted gene-wise regulative patterns as *weighted input vectors* over all test genes for each epigenome. Given ChromHMM emission vectors and ShallowChrome regulative patterns, we then proceed as follows (see the pipeline depicted in Supplementary Figure S4, Additional file 1): (1) *State Matching*: we associate each test gene with the chromatin state whose emission pattern maximises the Pearson’s correlation with the extracted ShallowChrome one (i.e., the *cosine similarity* computed between the two mean-centered vectors); (2) *Activation Prediction*: for each test gene, we compute the output logit as $y^{g,e} = b^{e} + \sum _{i=1}^{m} \varvec{\psi }_{i}^{g,e}$ (see Section “Model fitting and analysis” ); (3) *Gathering*: for each of the 15 considered chromatin states, we collect the output logits of all the matched genes and compute their mean; (4) *Ranking*: for each epigenome, we rank chromatin states according to their associated mean output logit. This gives us an informative overview on the expression characterisation our model assigns to such states, and indirectly allows us to validate our approach: matching our extracted regulative patterns with ChromHMM emission vectors gives them a semantic interpretation that can be verified a posteriori in terms of the associated gene activation rank. We remark that we study rankings instead of plain model logits so to be consistent with the model performance evaluation metric reported in the previous sections (AUROC).

**Fig. 4:**
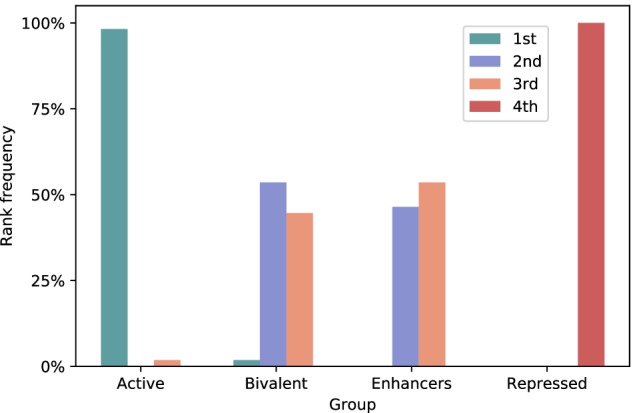
Aggregated ranking visualisation for chromatin state groups across the 56 considered epigenomes

In Fig. 4 we show an aggregated visualisation of the chromatin state rankings across the 56 considered epigenomes. To build such visualisation, chromatin states were clustered into 4 semantically consistent groups, and ranks were directly computed at this coarser level. This grouping was done to account for the inherent resolution gap between the ChromHMM and ShallowChrome models: the former one is trained to perform inference over 15 distinct chromatin characterisations at the level of 200 bp-long segments; the latter one is optimised to solve the *binary* classification of transcription states from an aggregated gene-wise signal spanning 10k bp (the group assignments as well as the ranking finer visualization are, respectively, in Supplementary Table S8 and Figure S5, Additional file 1).

In Fig. 4, for each group we report a histogram summarising the ranks scored across epigenomes. We observe that ShallowChrome strongly agrees with ChromHMM. The “Active” group, which includes all states associated with active transcription, attains the highest rank in all except one epigenome; whereas the “Repressed” group, which gathers all the states of transcriptionally inactive chromatin, always attains the lowest possible rank. Finally, the “Bivalent” chromatin and “Enhancers” groups show variability in the attained ranks, generally scoring in between activated and repressed states. This is exactly what is expected for the “Bivalent” group, where histone marks of activation and repression coexist to represent currently repressed genes, which have however a pre-assembled transcription machinery on their promoters, and therefore able to activate in very short time. This result has a different interpretation in the case of enhancer marks, which are distal regulation sites, whose influence is looser than that of the corresponding gene promoters, and therefore can tolerate a higher variability in the levels of the histone marks.

## Discussion

Throughout this work we showed how, building on top of established tools such as ‘peak calling’ algorithms, it is possible to model epigenetic trancriptional regulation meeting both the desiderata of accuracy and interpretability. The predictive power of the features extracted by our approach allows to achieve state-of-the-art results with well-studied statistical models (logistic regression), which can be directly inspected.

A relevant contribution of our work therefore lies in demonstrating how the application of complex deep learning modeling approaches may not always be the best choice, especially in problems where inductive biases are less intuitively evident, clear model interpretation is critical, and valuable domain-specific analysis and pre-processing tools are available. We believe that different modeling approaches could be more or less suitable depending on the specifics of the task at hand. Aiming at achieving an optimal trade-off between model bias and variance, their choice must be driven by a careful understanding of the problem and the available data (quantity, diversity, resolution).

Although deep learning architectures may not represent the best approach for the specific problem studied in this work, their application could be, on the contrary, more appropriate in other settings. For example, these techniques have been successfully applied to predict gene expression directly from the genomic sequence, such as the model in [36]. Other works include [37], where a sequence-based attention model additionally predicts chromatin accessibility, [38], where long range interactions between promoters and distant (non-coding) DNA sequences are considered, and [39], which further studies relational information in the form of promoter-enhancers associations. We note that many of these works could potentially be integrated with our ShallowChrome pipeline.

Finally, we also remark the importance of modeling assumptions and problem setting: in all cases where, as in this work, a prediction task is instantiated to indirectly study the relation between variables involved in a modeling effort, it is of paramount importance to take all choices made in formulating the task into consideration to contextualize any result and emergent conclusion. Within the scope of this work, a relevant role is, for example, held by the choice of a proper threshold to define binary classes. Throughout the presented analyses, the threshold has been set according to the per-tissue median RPKMs, in accordance with previous state-of-the-art models we compare with. In Supplementary Section S5, Additional file 1 , we further study the impact of this modeling choice by proposing an alternative approach based on detecting a local ‘valley’ between low and high-measurements in the empirical distribution of mRNA abundance levels. This alternative approach leads a linear model, such as ShallowChrome, to improve test results over all epigenomes, suggesting how artificial choices in the problem formulation, such as the binarisation threshold, may, in fact, implicitly encourage the use of more complex, nonlinear models. We envision to deepen these considerations and to analyse the resulting conclusions in future developments of this work.

## Conclusion

In this work we presented ShallowChrome, a novel computational pipeline to model accurately and in a fully interpretable way the transcriptional regulation layer controlled by histone mark modifications. The core stage of the pipeline consists of a scalable feature extraction step that, building on top of the well-established procedure of ‘peak calling’, allows retrieving gene-wise, significant and dynamically located epigenetic signal features for each of the considered regulators. The quality of the extracted features is confirmed by the largely satisfying experimental results obtained by simple logistic regression models on the task of binary classification of gene transcription states when such features are considered as input signals: our approach significantly outperforms recent state-of-the-art deep learning models on almost all of the 56 considered epigenomes. Finally, and most importantly, by employing generalized linear models our approach is inherently interpretable: each single parameter can be inspected in terms of sign, magnitude and even statistical significance, and corresponding gene-wise patterns can be easily derived to shed light on the role of histone marks in regulating the transcription of specific genes. In light of our results, we envision our accessible approach to find immediate and relevant application in the study of epigenetic transcriptional regulation within the research community.

## Supplementary Information

**Additional file 1.** Supplementary materials for “Accurate and highly interpretable prediction of gene expression from histone modifications”. **S1**. Considered epigenomes and classification performance. **S2**. Hyperparameter search space.** S3**. Sensitivity analyses. **S4**. Validation against ChromHMM chromatin states. **S5**. An alternative approach to binary thresholding.** S6**. ShallowChrome as a regression model. **S7**. Cross-epigenome generalisation.** S8**. Reproducing AttentiveChrome results.

## Data Availability

The datasets generated and analysed during the current study are available in the Zenodo repository at https://zenodo.org/record/4445287. At https://github.com/DEIB-GECO/ShallowChrome the Python source code of the implementation of the described ShallowChrome method is available, together with Jupyter notebooks to reproduce all paper results.

## Abbreviations

AUPR: Area under precision recall curve
AUROC: Area under receiver operating characteristic curve
bp: Base pair
ChIP: Chromatin ImmunoPrecipitation
GMQL: GenoMetric Query Language
HM: Histone mark modification
NGS: Next-generation sequencing
PAX5: PAired boX 5
REMC: Roadmap epigenomics mapping consortium
RPKM: Reads-per-kilobase-million
TSS: Transcription start site
TTS: Transcription termination site

## Footnotes

1 http://www.bioinformatics.deib.polimi.it/GMQLsystem/.

2 https://egg2.wustl.edu/roadmap/web_portal/chr_state_learning.html#core_15state

## References

[1] Phillips T (2008). Regulation of transcription and gene expression in eukaryotes. Nature Educ.

[2] van Steensel B (2011). Chromatin: constructing the big picture. EMBO J.

[3] Bannister A, Kouzarides T (2011). Regulation of chromatin by histone modifications. Cell Res.

[4] Bannister A, Kouzarides T (2005). Reversing histone methylation. Nature.

[5] Bradbury EM (1992). Reversible histone modifications and the chromosome cell cycle. BioEssays.

[6] Patnaik A (2019). Drugs targeting epigenetic modifications and plausible therapeutic strategies against colorectal cancer. Front Pharmacol.

[7] Miller JL, Grant PA (2013). The role of DNA methylation and histone modifications in transcriptional regulation in humans. Subcell Biochem.

[8] Sodersten E (2018). A comprehensive map coupling histone modifications with gene regulation in adult dopaminergic and serotonergic neurons. Nat Commun.

[9] Zhang L (2018). Revealing transcription factor and histone modification co-localization and dynamics across cell lines by integrating ChIP-seq and RNA-seq data. BMC Genomics.

[10] Singh R (2016). DeepChrome: Deep-learning for predicting gene expression from histone modifications. Bioinformatics.

[11] Singh R (2017). Attend and predict: Understanding gene regulation by selective attention on chromatin. Adv Neural Inf Process Syst.

[12] Ernst J, Kellis M (2017). Chromatin-state discovery and genome annotation with ChromHMM. Nat Protoc.

[13] Karlic R (2010). Histone modification levels are predictive for gene expression. Proc Natl Acad Sci USA.

[14] Costa I (2011). Predicting gene expression in T cell differentiation from histone modifications and transcription factor binding affinities by linear mixture models. BMC Bioinformatics.

[15] Cheng C (2016). A statistical framework for modeling gene expression using chromatin features and application to modENCODE datasets. Genome Biol.

[16] Dong X (2012). Modeling gene expression using chromatin features in various cellular contexts. Genome Biol.

[17] Sekhon A (2018). DeepDiff: DEEP-learning for predicting DIFFerential gene expression from histone modifications. Bioinformatics.

[18] Bahdanau D, et al. Neural machine translation by jointly learning to align and translate. In International Conference on Learning Representations, 2015;1–15.

[19] Kundaje A (2015). Integrative analysis of 111 reference human epigenomes. Nature.

[20] Feng J (2012). Identifying ChIP-seq enrichment using MACS. Nat Protoc.

[21] Kent W (2002). The human genome browser at UCSC. Genome Res.

[22] Kim A-Y (2015). The TFG-TEC oncoprotein induces transcriptional activation of the human *β*-enolase gene via chromatin modification of the promoter region. Mol Carcinog.

[23] Sharifi-Zarchi A (2017). DNA methylation regulates discrimination of enhancers from promoters through a H3K4me1-H3K4me3 seesaw mechanism. BMC Genomics.

[24] Lomvardas S, Thanos D (2002). Modifying gene expression programs by altering core promoter chromatin architecture. Cell.

[25] Lundberg SM, Lee S-I (2017). A unified approach to interpreting model predictions. Adv Neural Inf Process Syst.

[26] Lu Y (2018). DeepPINK: reproducible feature selection in deep neural networks. Adv Neural Inf Process Syst.

[27] Bernstein B (2010). The NIH Roadmap Epigenomics Mapping Consortium. Nat Biotechnol.

[28] Masseroli M (2015). GenoMetric Query Language: a novel approach to large-scale genomic data management. Bioinformatics.

[29] Masseroli M (2019). Processing of big heterogeneous genomic datasets for tertiary analysis of Next Generation Sequencing data. Bioinformatics.

[30] Cobaleda C (2007). Pax5 the guardian of B cell identity and function. Nat Immunol.

[31] Fuxa M, Busslinger M (2007). Reporter gene insertions reveal a strictly B lymphoid-specific expression pattern of Pax5 in support of its B cell identity function. J Immunol.

[32] Struhl K (1999). Fundamentally different logic of gene regulation in eukaryotes and prokaryotes. Cell.

[33] Hlady R (2018). Integrating the epigenome to identify novel drivers of hepatocellular carcinoma. Hepatology.

[34] Long M (2018). The miR-96 and RAR*γ* signaling axis governs androgen signaling and prostate cancer progression. Oncogene.

[35] Heyn P (2019). Gain-of-function DNMT3A mutations cause microcephalic dwarfism and hypermethylation of Polycomb-regulated regions. Nat Genet.

[36] Agarwal V, Shendure J (2020). Predicting mRNA abundance directly from genomic sequence using deep convolutional neural networks. Cell Rep.

[37] Zhou J (2018). Deep learning sequence-based ab initio prediction of variant effects on expression and disease risk. Nat Genet.

[38] Avsec Ž (2021). Effective gene expression prediction from sequence by integrating long-range interactions. Nat Meth.

[39] Zeng W, et al. Integrating distal and proximal information to predict gene expression via a densely connected convolutional neural network. Bioinformatics. 2019. 10.1093/bioinformatics/btz562. 10.1093/bioinformatics/btz562 31318408

