# Supplementary material for: Accurate and highly interpretable prediction of gene expression from histone modifications
Source: BMC Bioinformatics. 2022 Apr 26;23:151. doi: 10.1186/s12859-022-04687-x (PMC9040271; doi:10.1186/s12859-022-04687-x)
Supplement: Supplementary file 1 — Additional file 1. Supplementary materials for “Accurate and highly interpretable prediction of gene expression from histone modifications”. S1. Considered epigenomes and classification performance. S2. Hyperparameter search space. S3. Sensitivity analyses. S4. Validation against ChromHMM chromatin states. S5. An alternative approach to binary thresholding. S6. ShallowChrome as a regression model. S7. Cross-epigenome generalisation. S8. Reproducing AttentiveChrome results. [file 12859_2022_4687_MOESM1_ESM.pdf]

Supplementary materials for “Accurate and highly interpretable prediction of gene expression from histone modifications”

Fabrizio Frasca, Matteo Matteucci, Michele Leone, Marco J. Morelli and Marco Masseroli

S1 Considered epigenomes and classification performance

In Table S1 we report ShallowChrome test AUROC scores (*mean* ± *standard deviation*) on each of the epigenomes (i.e., cell-types/tissues) under study; we also report the fitted parameter values and the hyperparameter choice on the specific dataset split used in [1, 2].

Table S1: The 56 cell-types/tissues selected for this study from the REMC database [3] along with ShallowChrome AUROC scores (as *mean* ± *standard deviation* on test splits), fitted intercept (b), weights ($\mathbf{w}$) and estimated hyperparameter configuration (conf.) on the standard split utilised in [1, 2], as reported in Table S2. Parameters $\mathbf{w}$ are reported for histone modifications *H3K27me3*, *H3K36me3*, *H3K4me1*, *H3K4me3*, and *H3K9me3* (left to right), respectively.

REMC ID	Cell-type / Tissue (Epigenome)	AUROC Score	b	$\mathbf{w}$	conf.
E003	H1_Cell_Line	87.802 ± 0.300	**-0.900**	**-0.119, 0.186, 0.108, 0.101, -0.036**	#3
E004	H1_BMP4_Derived_Mesendoderm_Cultured_Cells	87.933 ± 0.444	-0.801	-0.198, 0.063, 0.070, 0.303, -0.008	#3
E005	H1_BMP4_Derived_Trophoblast_Cultured_Cells	88.435 ± 0.408	-0.810	-0.099, 0.069, 0.069, 0.049, -0.030	#1
E006	H1_Derived_Mesenchymal_Stem_Cells	87.191 ± 0.383	-0.615	-0.192, 0.206, 0.011, 0.037, -0.113	#4
E007	H1_Derived_Neuronal_Progenitor_Cultured_Cells	88.731 ± 0.363	-0.857	-0.143, 0.106, 0.033, 0.074, -0.037	#1
E011	hESC_Derived_CD184+_Endoderm_Cultured_Cells	87.402 ± 0.275	-0.820	-0.081, 0.230, 0.047, 0.056, -0.043	#4
E012	hESC_Derived_CD56+_Ectoderm_Cultured_Cells	89.788 ± 0.245	-0.961	-0.084, 0.172, 0.050, 0.034, -0.001	#5
E013	hESC_Derived_CD56+_Mesoderm_Cultured_Cells	89.117 ± 0.367	-0.596	-0.203, 0.213, 0.040, 0.016, -0.034	#4
E016	HUES64_Cell_Line	88.458 ± 0.319	-0.958	-0.084, 0.246, 0.071, 0.028, -0.034	#4
E024	4star	87.987 ± 0.384	-0.592	-0.304, 0.301, 0.010, 0.053, -0.017	#4
E027	Breast_Myoepithelial_Cells	86.807 ± 0.357	-0.750	-0.130, 0.181, -0.004, 0.028, 0.008	#5
E028	Breast_vHMEC	88.682 ± 0.294	-0.749	-0.046, 0.066, 0.064, 0.037, -0.012	#1
E037	CD4_Memory_Primary_Cells	89.127 ± 0.236	-0.632	-0.278, 0.232, 0.060, 0.076, -0.019	#4
E038	CD4_Naive_Primary_Cells	89.078 ± 0.253	-0.691	-0.351, 0.263, 0.046, 0.081, -0.027	#5
E047	CD8_Naive_Primary_Cells	89.828 ± 0.217	-0.613	-0.332, 0.207, 0.050, 0.062, -0.032	#5
E050	Mobilized_CD34_Primary_Cells_Female	90.460 ± 0.257	-0.608	-0.275, 0.215, 0.007, 0.035, -0.000	#4
E053	Neurosphere_Cultured_Cells_Cortex_Derived	89.307 ± 0.264	-0.834	-0.194, 0.258, 0.028, 0.019, -0.000	#4
E054	Neurosphere_Cultured_Cells_Ganglionic_Eminence_Derived	88.783 ± 0.155	-0.788	-0.220, 0.300, 0.020, 0.019, 0.007	#4
E055	Penis_Foreskin_Fibroblast_Primary_Cells_skin01	90.092 ± 0.387	-0.756	-0.093, 0.097, 0.013, 0.027, -0.054	#1
E056	Penis_Foreskin_Fibroblast_Primary_Cells_skin02	89.096 ± 0.330	-0.680	-0.109, 0.083, 0.018, 0.031, -0.094	#1
E057	Penis_Foreskin_Keratinocyte_Primary_Cells_skin02	88.246 ± 0.322	-0.817	-0.245, 0.352, 0.015, 0.026, -0.029	#4
E058	Penis_Foreskin_Keratinocyte_Primary_Cells_skin03	89.275 ± 0.285	-0.793	-0.078, 0.120, 0.003, 0.021, -0.017	#1
E059	Penis_Foreskin_Melanocyte_Primary_Cells_skin01	87.605 ± 0.274	-0.536	-0.172, 0.103, 0.062, 0.036, -0.056	#1
E061	Penis_Foreskin_Melanocyte_Primary_Cells_skin03	89.074 ± 0.373	-0.774	-0.106, 0.099, 0.030, 0.026, -0.009	#1
E062	Peripheral_Blood_Mononuclear_Primary_Cells	88.339 ± 0.187	-0.355	-0.330, 0.174, 0.067, 0.032, -0.079	#4
E065	Aorta	80.842 ± 0.337	-0.123	-0.158, 0.163, 0.070, 0.083, -0.141	#4
E066	Adult_Liver	84.975 ± 0.386	-0.317	-0.216, 0.153, 0.003, 0.049, -0.060	#4
E070	Brain_Germinal_Matrix	85.756 ± 0.287	-0.661	-0.190, 0.187, 0.044, 0.026, 0.052	#4
E071	Brain_Hippocampus_Middle	81.386 ± 0.426	-0.535	-0.137, 0.164, 0.060, 0.023, -0.086	#4
E079	Esophagus	84.087 ± 0.293	-0.571	-0.090, 0.072, 0.049, 0.025, -0.040	#1
E082	Fetal_Brain_Female	86.323 ± 0.229	-0.866	-0.151, 0.191, 0.048, 0.024, 0.015	#4
E084	Fetal_Intestine_Large	85.549 ± 0.407	-0.562	-0.133, 0.145, 0.049, 0.021, -0.030	#5
E085	Fetal_Intestine_Small	86.363 ± 0.426	-0.655	-0.061, 0.055, 0.045, 0.022, 0.005	#1
E087	Pancreatic_Islets	83.924 ± 0.243	-0.386	-0.214, 0.095, 0.044, 0.044, -0.084	#4
E094	Gastric	82.661 ± 0.302	-0.496	-0.173, 0.152, 0.040, 0.043, -0.159	#5
E095	Left_Ventricle	87.473 ± 0.366	-1.053	-0.212, 0.191, 0.262, 0.136, -0.149	#2
E096	Lung	84.089 ± 0.348	-0.461	-0.102, 0.076, 0.040, 0.043, -0.046	#1
E097	Ovary	84.009 ± 0.313	-0.030	-0.405, 0.195, 0.179, 0.113, -0.339	#2
E098	Pancreas	84.250 ± 0.230	-0.725	-0.098, 0.099, 0.063, 0.025, -0.087	#1
E100	Psoas_Muscle	88.465 ± 0.361	-0.686	-0.172, 0.116, 0.116, 0.059, -0.040	#1
E104	Right_Atrium	84.114 ± 0.367	-1.081	-0.321, 0.012, 0.371, 0.131, -0.036	#2
E105	Right_Ventricle	85.575 ± 0.328	-0.549	-0.167, 0.084, 0.027, 0.023, -0.047	#1
E106	Sigmoid_Colon	84.818 ± 0.438	-0.213	-0.267, 0.274, 0.062, 0.058, -0.179	#4
E109	Small_Intestine	83.271 ± 0.486	-0.597	-0.237, 0.344, 0.088, 0.058, -0.132	#4
E112	Thymus	83.361 ± 0.345	-0.406	-0.214, 0.269, 0.032, 0.024, -0.118	#4
E113	Spleen	85.408 ± 0.372	-0.590	-0.212, 0.136, 0.046, 0.071, -0.148	#5
E114	A549	89.235 ± 0.444	-0.885	-0.160, 0.138, 0.031, 0.071, 0.031	#1
E116	GM12878	90.511 ± 0.210	**-0.674**	**-0.269, 0.281, -0.015, 0.127, 0.054**	#4
E117	HELA	91.300 ± 0.341	-0.856	-0.141, 0.141, 0.007, 0.040, -0.016	#1
E118	HEPG2	90.319 ± 0.314	-0.849	-0.232, 0.337, 0.050, 0.041, -0.053	#4
E119	HMEC	89.433 ± 0.444	-0.618	-0.122, 0.093, 0.018, 0.038, -0.010	#1
E120	HSMM	89.197 ± 0.265	-0.701	-0.135, 0.120, 0.057, 0.032, -0.035	#1
E122	HUVEC	88.881 ± 0.467	-0.849	-0.180, 0.254, 0.149, 0.096, -0.088	#2
E123	K562	91.957 ± 0.168	**-0.930**	**-0.098, 0.153, 0.033, 0.081, -0.002**	#1
E127	NHEK	89.446 ± 0.443	-0.694	-0.182, 0.236, 0.028, 0.036, -0.002	#5
E128	NHLF	89.072 ± 0.450	-0.608	-0.203, 0.197, 0.029, 0.064, -0.025	#4

In particular, let us highlight the parameters learnt for cell-types E003, E116, E123 (boldtype in Table S1), for which we analysed the weighted input patterns for gene PAX5 in Subsection "Gene-wise regulative patterns" of the main manuscript. As it is possible to notice, they significantly differ from each other. This confirms that the distinct patterns observed for gene PAX5 across the three epigenomes are not simply due to a different histone modification behavior but, rather to the joint interplay between measured epigenetic activity and the epigenome-specific model parameters.

S2 Hyperparameter search space

The hyperparameter search space $\mathcal{H}$ we considered is reported in Table S2; it defines the peak calling output formats used for the histone modifications in our study. As it can be observed in the table, we did not search exhaustively on all possible format combinations. Besides the simple approach of choosing a uniform format across all epigenetic regulators (configurations #1 − #3), we also included hybrid configurations where H3K4me1 and H3K4me3 are assigned a narrower peak format than H3K36me3, H3K9me3 and H3K27me3. This is in accordance to the classical characterization such that the former ones are usually addressed as "TF-like marks", due to the fact that their ChIP signals usually exhibit peaks that are sharper and more localized around TSSs than the latter ones [4].

Table S2: Hyperparameter search space: Peak calling output formats used for the considered histone modification signals.

Configuration	H3K4me3	H3K4me1	H3K36me3	H3K9me3	H3K27me3
#1	NarrowPeak	NarrowPeak	NarrowPeak	NarrowPeak	NarrowPeak
#2	BroadPeak	BroadPeak	BroadPeak	BroadPeak	BroadPeak
#3	GappedPeak	GappedPeak	GappedPeak	GappedPeak	GappedPeak
#4	NarrowPeak	NarrowPeak	BroadPeak	BroadPeak	BroadPeak
#5	NarrowPeak	NarrowPeak	GappedPeak	GappedPeak	GappedPeak
#6	BroadPeak	BroadPeak	GappedPeak	GappedPeak	GappedPeak

S3 Sensitivity analyses

In this section we report additional analyses on the sensitivity of our approach to the choice of evaluation metric and input features.

S3.1 Additional evaluation metrics

For completeness, we report here test classification results in terms F1-score and Area Under Precision-Recall Curve (AUPR). These last ones represent other commonly used evaluation metrics, which may be preferred to the AUCROC metric under certain circumstances, such as skewness in the class distribution.

Table S3: Aggregated statistics on the test F1-scores for DeepChrome, AttentiveChrome and ShallowChrome computed across the 56 considered epigenomes. Last column: aggregated statistics on the test AUPR for ShallowChrome.

Statistic	DeepChr.	Att.Chr.	Shall.Chr.	Shall.Chr. (AUPR)
Mean	0.5500	0.5600	0.8156	0.8470
Median	0.6900	0.6200	0.8229	0.8569
Max	0.8900	0.8800	0.8695	0.8989
Min	0.1200	0.1600	0.7560	0.7746

Table S3 reports mean, median, max and min average F1-scores over all the 56 epigenomes considered in the study, for the hyperparameters previously estimated to maximise validation AUROC (they have not been re-tuned to optimise for F1-score). On average, ShallowChrome significantly outperforms the AttentiveChrome and DeepChrome deep learning baselines also according to this additional performance metric. In particular, as it is possible to observe from the table, ShallowChrome does not exhibit the peculiar performance drop that instead characterises the baseline models over certain

epigenomes. This behavior is already discussed in the main text w.r.t. test AUROC, and is here additionally validated in terms of test F1-score.

The same Table S3 reports, in the last column, mean, median, max and min average test AUPR-scores, again for the hyperparameters maximising validation AUROC. The results remain in line with those reported for the other AUROC and F1-Score metrics; and we do not observe any noteworthy drop in performance. Unfortunately, no results in terms of AUPR are reported in the original AttentiveChrome and DeepChrome manuscripts. Nevertheless, we managed to reproduce AttentiveChrome test predictions on almost all epigenomes, by running pretrained models provided by the authors. This allowed to compute AUPR scores for this model, which are reported in Section S8.

Finally, in Figure S1 we additionally report the confusion matrices obtained by ShallowChrome over the test sets of cell lines H1-hESC, GM12878 and K562. The relative proportions of True Positives, False Positives, True Negatives and False Negatives are all consistent with the performance previously analysed in terms of the other evaluation metrics.

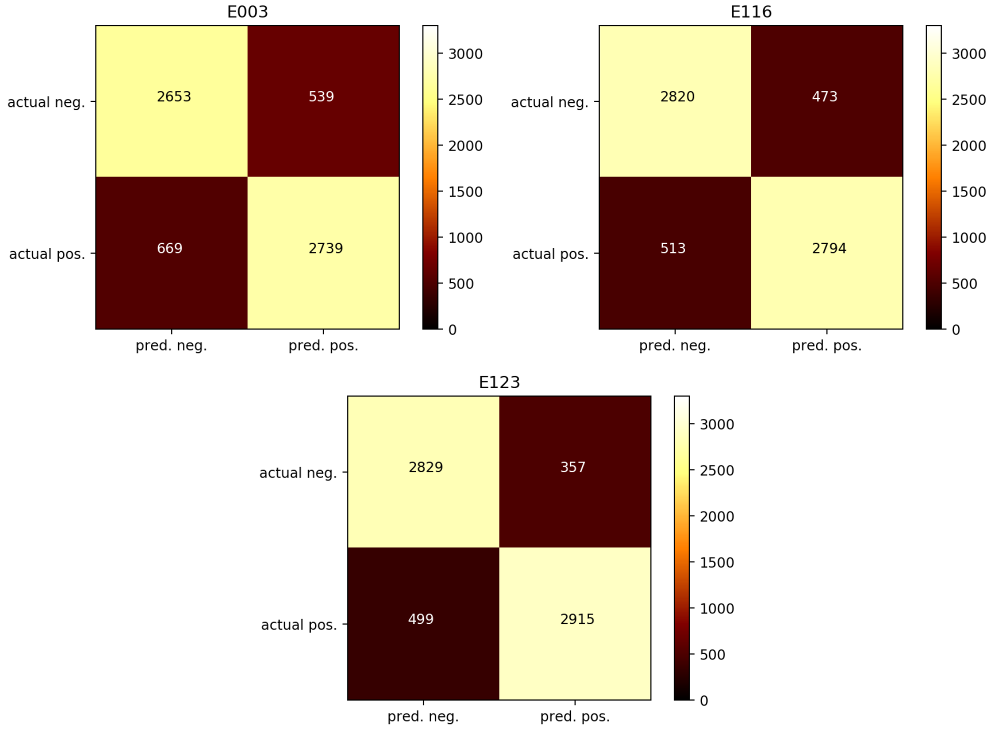

Figure S1: Test confusion matrices for epigenomes ‘E003’ (H1-hESC), ‘E116’ (GM12878), ‘E123’ (K562).

S3.2 Choice of input signals

Here, we study how model performance is affected by employing only a subset of markers. Indeed, in certain situations, researchers may not possess ChIP-seq signals for all the 5 histone modifications considered in this study, for a certain cell-type/tissue of interest. In our work, the choice of markers has been driven by the necessity to fairly compare with previous works, where these have been selected mostly due to their uniform profiling over the considered cell-types/tissues. Nonetheless, we remark that the presented ShallowChrome pipeline is independent on the specific selection of histone modifications, and that accurate results can still be obtained with smaller sets of epigenetic signals.

In Figure S2 we report the test performance obtained by only considering subsets of markers over the cell lines H1-hESC, GM12878 and K562.

As expected, best results are obtained when employing the complete set of markers. However, we notice that strong performance is obtained by only using the signal from *H3K4me3*; this is expected as this marker typically indicates the presence of open chromatin over promoter regions. Lastly, we interestingly notice how the worst results are obtained exactly when only using *H3K27me3*, which typically marks regions of repressed transcription. As expected, this marker alone is not sufficient to accurately predict a status of active transcription.

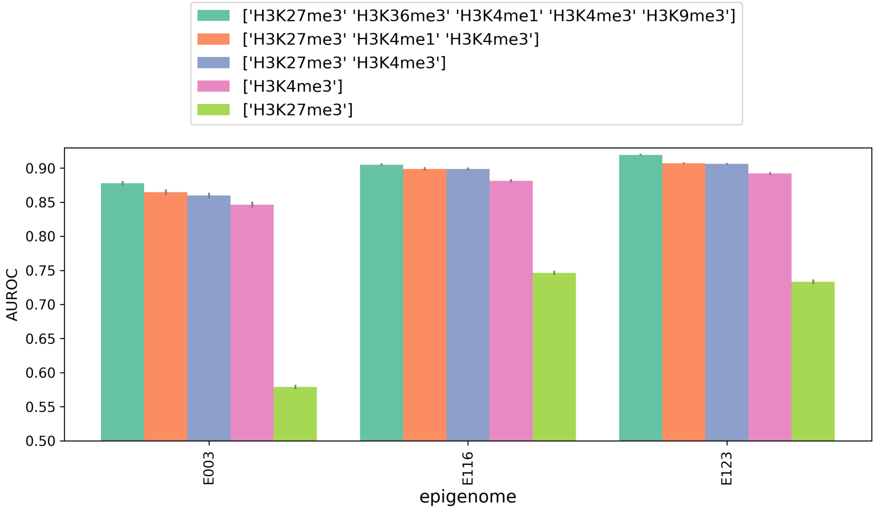

Figure S2: Test AUROC of ShallowChrome trained with only subsets of markers (reported in legend).

S3.3 Intercept parameter

Throughout our experimental section, we have evaluated the method reporting its performance over different cell-types/tissues and binary class thresholding based on the median expression value. In this setting it may be hypothesised that the model intercept may play a substantial and possibly predominant role in obtaining competitive results. We additionally studied this aspect by fitting ShallowChrome models with no access to an experiment-wide intercept parameter. Results are reported in Figure S3 and Table S4.

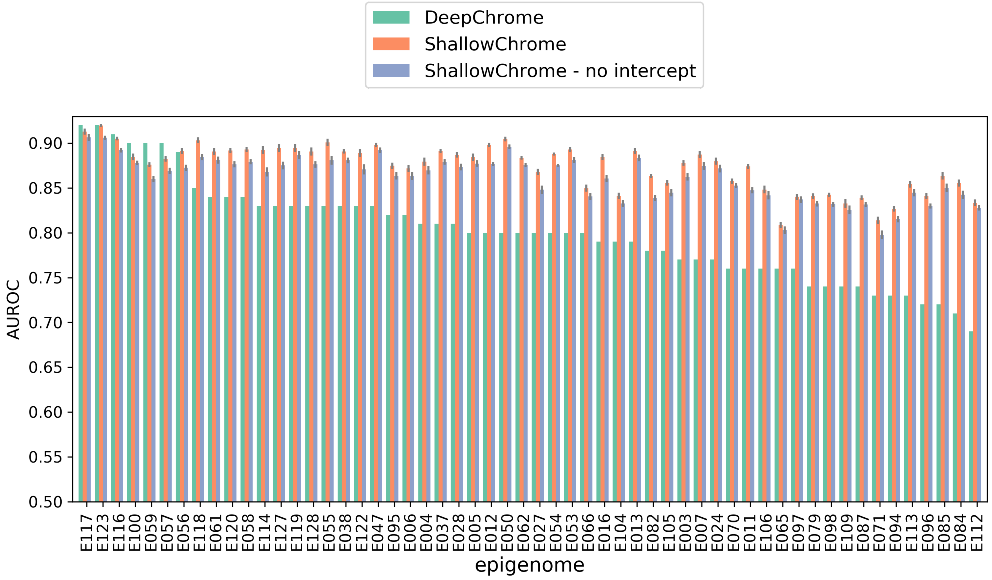

Figure S3: Test AUROC scores for DeepChrome, ShallowChrome and ShallowChrome trained without intercept. Results are reported on the 56 considered epigenomes, which are indicated with their respective REMC code (see Table S1 for the association between REMC codes and epigenomes).

As expected, test performance generally decreases when not using an intercept parameter. However, the average drop is contained in all result statistics (around ∼1.2% in absolute terms), and ShallowChrome still largely outperforms deep learning baselines. This evidently demonstrates that, although the intercept parameter contributes to the competitive performance of our method, yet it does not drive the entirety of it.

On a side note, we remark here that, as already observed in the main text, the intercept may

Table S4: Aggregated statistics on the test AUROC scores for DeepChrome, AttentiveChrome, ShallowChrome, ShallowChrome trained without intercept. These have been computed across the 56 considered epigenomes.

Statistic	DeepChr.	Att.Chr.	Shall.Chr.	Shall.Chr. (no int.)
Mean	0.8008	0.8133	0.8737	0.8613
Median	0.6900	0.6200	0.8829	0.8694
Max	0.9225	0.9218	0.9196	0.9063
Min	0.6854	0.7237	0.8084	0.7978

convey interesting information on the transcriptional state of genes for which no histone modification activity is measured.

S3.4 Input window length

We defined as *input-fields* those gene-associated genomic locations where signals in input to our data extraction pipeline are considered. In particular, in accordance with the choice made in [1, 2], we chosen *input-fields* as symmetric 10k bps windows centered on each gene TSS.

In order to assess the impact of such a design choice, we also experimented with symmetric windows of length 2k, 4k, 6k, 8k bps. Results are reported in Table S5 in terms of aggregated statistics on the mean test AUROC, across epigenomes.

Table S5: Aggregated statistics on ShallowChrome test AUROC scores for different choices of the input window length (in bps); the former ones have been computed across the 56 considered epigenomes.

Statistic	10k	8k	6k	4k	2k
Mean	0.8737	**0.8745**	0.8742	0.8726	0.8666
Median	0.8829	**0.8834**	0.8828	0.8804	0.8729
Max	0.9196	0.9207	**0.9216**	0.9200	0.9154
Min	0.8084	**0.8099**	0.8079	0.8043	0.7972

As it is possible to observe, the length of the input-field does not significantly impact test performance. We generally report rather marginal variations, with best results obtained by the intermediate 6k and 8k bp values, and worst results obtained by the shortest 2k-long windows. We hypothesise these last may miss predictive epigenetic signals located farther from the TSS.

Interestingly, 6k-long windows appear to work particularly better than the standard 10k ones on a particular epigenome: E097; we report a test AUROC of 0.8514 ± 0.0038 vs. 0.8401 ± 0.0031. Specific analyses on this sample will be object of future endeavors.

S3.5 Aggregation statistic

ShallowChrome selects the *max* peak score over *input-fields* in the *Extraction* phase (see Method section "Feature extraction" in main manuscript). However, the whole pipeline is fully compatible with the choice of other aggregators. In particular, we conducted additional experiments to study the impact of other aggregation functions: *sum*, *mean*, *min*. Results are reported in Table S6 in terms of aggregated statistics on the mean test AUROC, across epigenomes.

Table S6: Aggregated statistics (Stat.) on ShallowChrome test AUROC scores for different choices of the aggregation measure (Aggr.); the former ones have been computed across the 56 considered epigenomes.

Stat. ↓ / Aggr. →	*max*	*sum*	*mean*	*min*
Mean	**0.8737**	0.8720	0.8651	0.8584
Median	**0.8829**	0.8785	0.8750	0.8656
Max	**0.9196**	0.9146	0.9163	0.9132
Min	0.8084	**0.8114**	0.8000	0.7965

We note that the *max* aggregator is the one that, in general, works best. As expected, *min* is the aggregator that generally performs worse, while *mean* and, in particular, *sum* are close in performance

to *max*. In particular, the *sum* aggregator attains the best minimum AUROC, and seems to work better on specific epigenomes, such as E098 (0.8510 ± 0.0025 vs. 0.8425 ± 0.0023). This may suggest that in some samples, epigenetic activity may be better modeled by jointly considering all events observed in the proximity of TSSs, in an additive fashion. This, rather then the 'strongest' measured event, may better predict gene expression states in some cases.

S3.6 Classifier capacity

Are the input features extracted by the ShallowChrome pipeline tight to the form of the classification model? In other words, can we employ a more capable classifier than simple logistic regression (LR)? In Table S7 we report the test results obtained by a more expressive model: a Multi-Layer-Perceptron (MLP) with one hidden layer consisting of 100 neurons (and Rectified Linear Units as activation functions).

Table S7: Aggregated statistics on ShallowChrome test scores for Logistic Regression (LR) and Multi-Layer-Perceptron (MLP) classifiers. These have been computed across the 56 considered epigenomes.

Statistic	AUROC		F1-Score		AUPR	
	LR	MLP	LR	MLP	LR	MLP
Mean	0.8737	0.8766	0.8156	0.8212	0.8470	0.8512
Median	0.8829	0.8847	0.8229	0.8275	0.8569	0.8614
Max	0.9225	0.9224	0.8695	0.8710	0.8989	0.9029
Min	0.8084	0.8108	0.7560	0.7659	0.7746	0.7755

We observe slight performance improvements from the use of a more expressive classifier across all metrics. However, it is the belief of the authors that these are not significant enough to outweigh the possibility to easily and directly interpret the parameters of the Logistic Regression model. These results confirm that, rather than the model itself, it is the specific feature selection strategy that mostly contributes to the high accuracy of the overall pipeline.

S4 Validation against ChromHMM chromatin states

S4.1 ShallowChrome pattern validation pipeline

We report in Figure S4 a cartoon depicting the ShallowChrome pattern validation pipeline, in its four distinct phases.

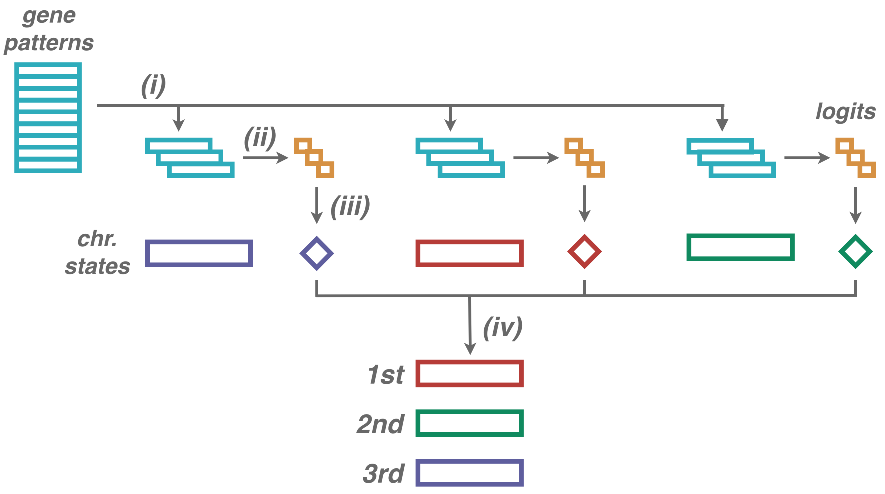

Figure S4: ShallowChrome pattern validation pipeline. Phases: (i) State Matching, (ii) Activation Prediction, (iii) Gathering, (iv) Ranking.

S4.2 ChromHMM state grouping and rankings

Table S8 describes how chromatin states were assigned to the 4 groups considered in the main text. The grouping allowed us to account for the inherent resolution gap between ChromHMM and ShallowChrome: in the former model, input signals are considered at a finer resolution level and inference is performed on a much larger number of chromatin states.

Table S8: Grouping of ChromHMM chromatin states.

Chromatin State	Group
Transcr. at gene 5' and 3'	Active
Active TSS	Active
Weak Transcription	Active
Strong Transcription	Active
Flanking Active TSS	Active
Bivalent/Poised TSS	Bivalent
Flanking Bivalent TSS/Enh	Bivalent
Bivalent Enhancers	Bivalent
Genic Enhancers	Enhancers
Enhancers	Enhancers
Weak Repressed PolyComb	Repressed
Repressed PolyComb	Repressed
Heterochromatin	Repressed
Quiescent/Low	Repressed
ZNF genes & repeats	Repressed

Here, in Figure S5 we report a visualization of the ranking finer than that in the main text: for *each chromatin state* we calculate its rank in each epigenome and then we box-plot all the computed ranks, with chromatin states sorted according to their median rank.

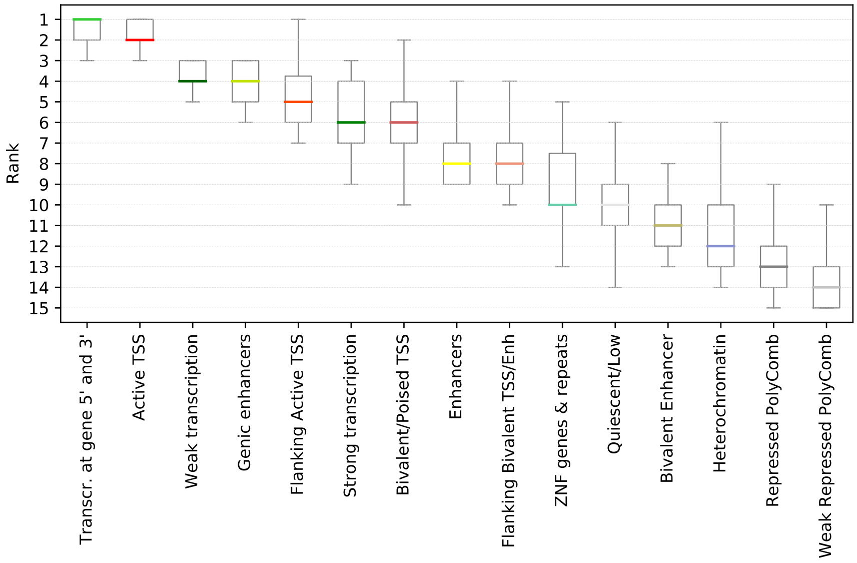

Figure S5: Aggregated ranking visualisation for *each chromatin state* across the 56 considered epigenomes. Outliers are excluded from boxplots to ease visualisation.

From the figure it is evident how predictions from our model still consistently recapitulate the expression levels generally associated with chromatin states. States associated with active transcription ("Transcr. at gene 5' and 3'", "Active TSS", "Weak transcription", "Flanking Active TSS", "Strong transcription") have all a tendency to score higher in the ranking (positions 1 to 6), i.e., to match regulative patterns of genes that are confidently predicted as 'ON'. On the contrary, states associated with inactive transcription ("ZNF genes & repeats", "Quiescent/Low", "Heterochromatin", "Repressed PolyComb", "Weak Repressed PolyComb") generally score lower in the ranking (positions 10 to 15).

Coherently with what already observed in main text, we notice that states for bivalent/poised chromatin ("Bivalent/Poised TSS", "Flanking Bivalent TSS/Enh") tend to place halfway, together with those indicating enhancers (positions 4 to 12).

We finally observe that this fine chromatin characterization introduces noise in terms of ranking uncertainty and of relative positions: as an example, and somehow counter-intuitively, the state "Weak Transcription" has a higher median rank than the state "Strong Transcription", which also exhibits larger ranking variations across epigenomes (see inter-quartile range in boxplot). This observation confirms our intuition that, although being an effective and interpretable approach to model epigenetic transcriptional regulation at the level of genes, ShallowChrome may not be the most appropriate methodology to capture chromatin aspects at such a fine resolution level, considering the used input signal.

S4.3 Direct matching of ShallowChrome inputs

We conclude this section by showing, for completeness, the resulting ranking obtained by directly matching ShallowChrome inputs downstream the feature extraction phase with ChromHMM chromatin states. The final ranking is depicted in Figure S6, obtained by following the pipeline depicted in Figure S4.

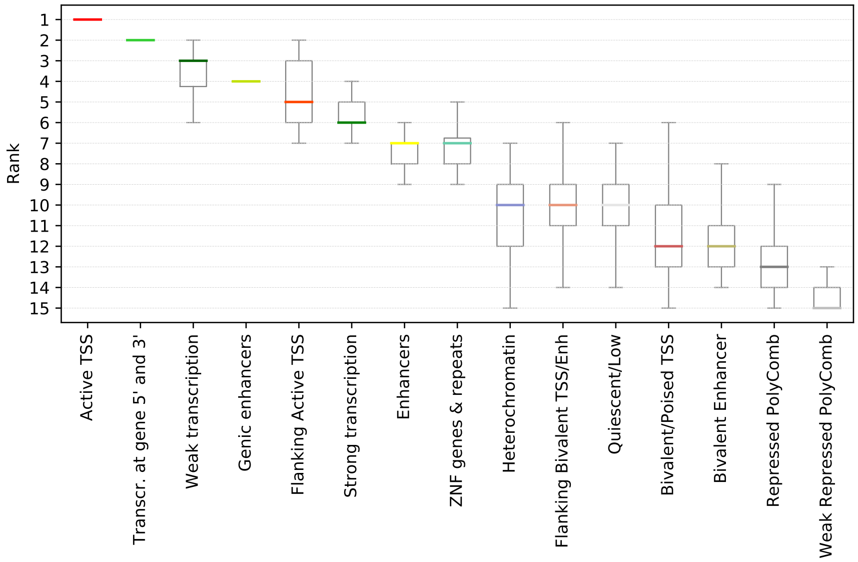

Figure S6: Aggregated ranking visualisation for *each chromatin state* across the 56 considered epigenomes. In this case, rankings have been obtained by directly matching inputs to ChromHMM chomatine states. Again, outliers are excluded from boxplots to ease visualisation.

We observe how the ranking still reasonably recapitulates the expression levels generally associated with chromatin states. Some differences w.r.t. the previous ranking in Figure S5 are reported; in particular, state 'Heterochromatin' is associated with a higher median rank and states in the lower region of the ranking are associated with slightly larger variance. Overall the ranking produced by weighted input patterns appear more robust, this confirming the important contribution of fitted model weights in generating valid patterns for interpretable explanations.

S5 An alternative approach to binary thresholding

In this section we propose an alternative approach to deriving binary classes from continuous quantifications of mRNA transcript abundances. Throughout our work, in accordance to [1, 2], we applied the binary thresholding method described in the main text, with an epigenome-specific threshold defined as the median mRNA quantification value. This approach relies on a straightforward assumption: a similar amount of active and inactive genes are present in each epigenome. Employing the median as a binary threshold, however, may assign to negative class ('OFF') also active genes with weaker transcriptional activity, therefore introducing noise in the supervision signal.

When the task is to distinguish transcriptionally active from inactive genes, we could also compute the threshold inducing a stark contrast between the two, without relying on the assumption described above. We hypothesize that the distribution of mRNA quantifications is the mixture of two distinct components: an exponential distribution, monotonically decreasing from 0.0 RPKMs and constituted by inactive genes and mostly representing Poissonian-distributed shot noise, and a Gaussian one, constituted by real contributions from active genes. Accordingly, a threshold to distinguish active from inactive genes has to be chosen to maximally separate these two components. We propose to take the threshold as the value corresponding to the local minimum between the two distributions. Visually, this corresponds to the lowest value in the characteristic "valley" emerging therein when plotting the empirical target distribution in log-scale. In Figure S7, we illustrate the thresholds obtained by the two methods on epigenome E071 ("Brain_Hippocampus_Middle"), i.e., the 'valley' (blue) and median (red) thresholds. The discrepancy between the two approaches is rather large, which may yield to large differences in both model fitting and evaluation.

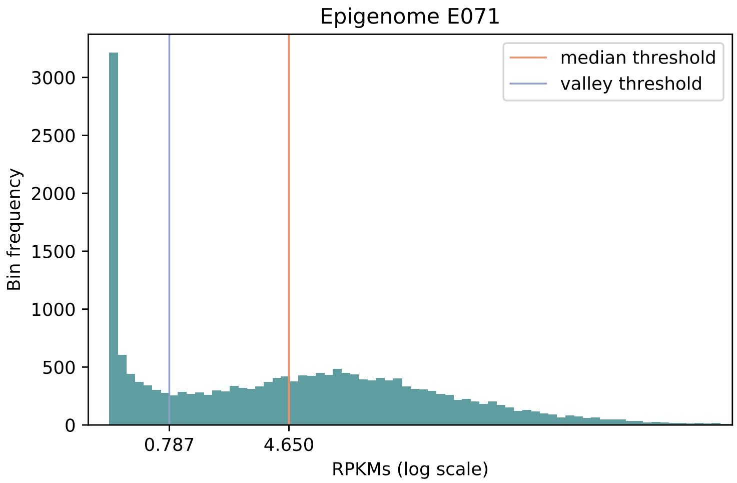

Figure S7: Binned distribution of log-transformed transcription quantifications for epigenome E071, along with its median and 'valley' thresholds.

We investigated the impact of this alternative approach by training and evaluating ShallowChrome with binary classes derived from the proposed binarisation. When adopting a non-median based thresholding scheme the relative percentage of positive and negative samples (genes) may vary, making the task unbalanced. We have therefore experimented with two scenarios: one in which classes are left unbalanced, and the other in which classes have been balanced following a simple subsampling scheme.

Table S9: Aggregated statistics on the AUROC, F1, AUPR test scores for ShallowChrome trained with standard median class thresholding and 'valley based' thresholding, in both a balanced and unbalanced setting. All statistics have been computed across the 56 considered epigenomes.

Statistic	AUROC			F1-Score			AUPR		
	median	vall., bal.	vall., unbal.	median	vall., bal.	vall., unbal.	median	vall., bal.	vall., unbal.
Mean	0.8737	0.8951	0.8961	0.8156	0.8340	0.8626	0.8470	0.8793	0.9103
Median	0.8829	0.8959	0.8964	0.8229	0.8347	0.8630	0.8569	0.8824	0.9120
Max	0.9196	0.9277	0.9274	0.8695	0.8740	0.8896	0.8989	0.9161	0.9433
Min	0.8084	0.8527	0.8492	0.7560	0.7888	0.8174	0.7746	0.8335	0.8531

In Table S9 and Figure S8 we report the test performance obtained when using "valley" thresholds. In particular, Table S9 additionally report F1 and AUPR metrics. It is interesting to notice how results are, overall, all significantly better than those obtained with the standard median approach adopted in the rest of this work and, in particular, ShallowChrome mean test scores are higher on all epigenomes. This remarkable improvement reflects how the new thresholds have made the two classes more linearly separable, smoothing out noise in the supervision signal; this last is likely to be overfitted by more capable models, easily driven to poor generalization on held out sets.

Finally, for completeness, we report in Table S10 the number of samples (genes) in each of the two classes after valley thresholding, along with the percentage of positives in the dataset.

Table S10: Details on class balance after valley thresholding.

Epigenome	# Positives	# Negatives	Positive ratio
E003	12604	7198	64%
E004	11527	8275	58%
E005	11340	8462	57%
E006	11660	8142	59%
E007	11939	7863	60%
E011	11466	8336	58%
E012	11226	8576	57%
E013	11675	8127	59%
E016	11733	8069	59%
E024	11401	8401	58%
E027	10189	9613	51%
E028	10212	9590	52%
E037	10711	9091	54%
E038	10884	8918	55%
E047	9984	9818	50%
E050	11069	8733	56%
E053	11895	7907	60%
E054	11770	8032	59%
E055	11351	8451	57%
E056	11030	8772	56%
E057	10563	9239	53%
E058	11265	8537	57%
E059	10879	8923	55%
E061	11121	8681	56%
E062	11129	8673	56%
E065	13104	6698	66%
E066	12209	7593	62%
E070	11604	8198	59%
E071	14266	5536	72%
E079	13034	6768	66%
E082	11844	7958	60%
E084	12411	7391	63%
E085	12990	6812	66%
E087	13501	6301	68%
E094	12289	7513	62%
E095	11998	7804	61%
E096	12517	7285	63%
E097	11389	8413	58%
E098	13952	5850	70%
E100	10195	9607	51%
E104	12171	7631	61%
E105	11987	7815	61%
E106	13009	6793	66%
E109	13491	6311	68%
E112	12892	6910	65%
E113	11627	8175	59%
E114	10656	9146	54%
E116	10594	9208	53%
E117	11229	8573	57%
E118	10965	8837	55%
E119	11266	8536	57%
E120	11306	8496	57%
E122	10250	9552	52%
E123	10743	9059	54%
E127	11105	8697	56%
E128	11214	8588	57%

Whilst all results presented in the main text have been obtained with standard median thresholding in order to compare with the state-of-the-art methods in [1, 2], we believe this alternative approach to deriving binary classes to be particularly promising; further analyses on the epigenetic regulative patterns extracted by the alternative fitting of ShallowChrome would help determining whether it can robustly replace other approaches adopted in the community so far.

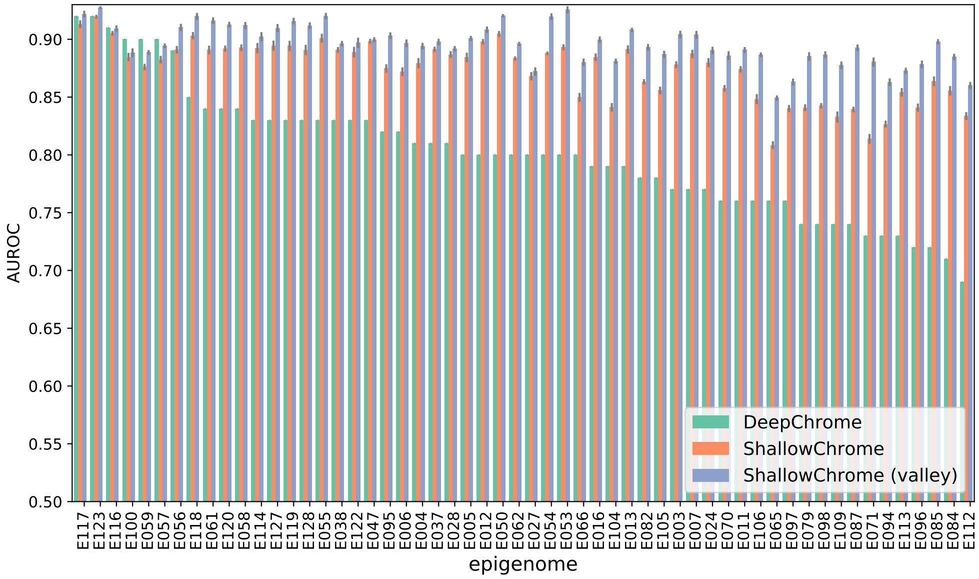

Figure S8: Test AUROC scores for DeepChrome, ShallowChrome and ShallowChrome with 'valley based' thresholding. Results are reported on the 56 considered epigenomes, which are indicated with their respective REMC code (see Table S1 for the association between REMC codes and epigenomes).

S6 ShallowChrome as a regression model

It is in the reference works of [1, 2] that the prediction of gene expression from histone modification activity has originally been cast as a binary classification problem; we have adopted the same problem setting throughout our work, in an effort to guarantee fair comparison of experimental results. However, the ShallowChrome modeling pipeline is completely compatible with other predictive settings. In particular, we can fit our linear model in a regression fashion, seeking to directly predict the real-valued measured mRNA abundance quantifications. Fitted models could then be inspected and interpreted exactly as presented in the main section of this manuscript, that is by means of weighted input patterns and statistical tests on the estimated values of weight parameters.

As an additional study, we retrained ShallowChrome in a regression fashion, optimising Mean Squared Error (MSE) loss between predictions and real mRNA abundance measurements. Inputs into the linear regression model are exactly the same employed in binary classification (max peak values attained in a 10k bps symmetric window over TSSs). As for target values, they were first processed by applying a log - square root transformation to reduce the impact of multiplicative noise and outliers, in accordance with previous works [5]. Results are reported in Table S11.

Table S11: ShallowChrome regression performance. Statistics are computed across epigenomes over mean coefficients of determination (R^2).

Statistic	ShallowChrome
Mean	0.5391
Median	0.5515
Max	0.6384
Min	0.3853

Involving punctual prediction of a real response value, regression is, per se, a much harder task

than classification on binarised targets. Nonetheless, ShallowChrome achieves reasonable performance across epigenomes. The median coefficient of determination of $R^2 = 0.5515$ demonstrates the importance of modeling input epigenetic features: they allow our model to significantly outperform the constant baseline predictor ($R^2 = 0.0$).

In order to better contextualise the above results, we compared model predictions obtained in a regression setting with predicted class probabilities from our classification models. We generally observed a strong correlation between these two quantities. In Figure S9, we plot them one against the other for cell lines E065 and E123, associated with, respectively, minimum and maximum obtained R^2 scores (see Table S11). In the plots, regression predictions are reported on the x-axis, distributed over 25 equally-sized bins. On the y-axis we report the mean predicted class probability for the genes on the corresponding bin (along with the standard deviation).

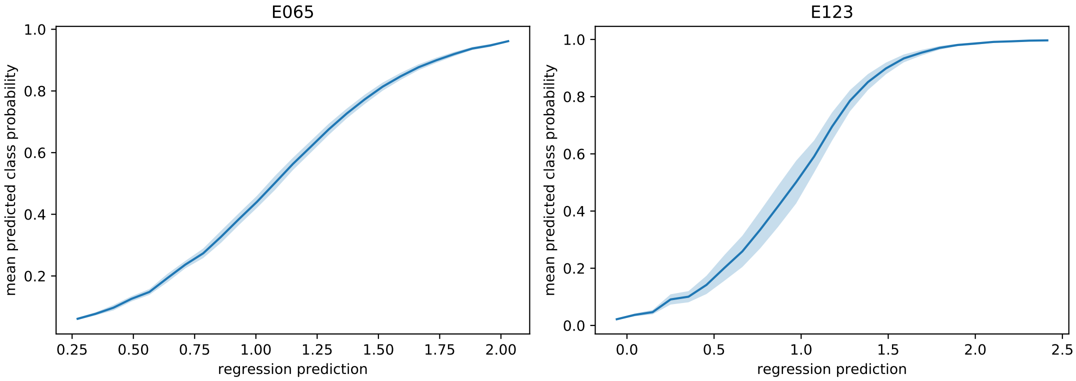

Figure S9: Test predictions from regression models against corresponding class probabilities from classification models over epigenomes E065 and E123.

As it can be observed, the overall trend well recapitulates the logistic activation of our classification models, confirming how predictions from our regression models are in strong accordance with predicted class probabilities. We note that these plots were realised by only considering test genes unseen at training time.

These additional analyses suggest how the ShallowChrome pipeline, which delivers highly accurate and interpretable predictions of gene activation state, can also provide valid indications of the actual gene expression level (in terms of mRNA abundance quantification); however, additional input signals (e.g., Transcription Factor binding, DNA methylation) may be required to achieve higher levels of accuracy for this prediction task.

S7 Cross-epigenome generalisation

In this section we apply the model trained on a specific cell type to another cell type to perform cross-cell-type predictions.

We fit our model on a given cell-type (that we label as 'source') and apply the same model to genes on cell lines whose epigenomes are considered 'similar' or 'different'. We consider two cases: (i) source: *H1 Cell Line* (E003), similar: *H1 BMP4 Derived Trophoblast Cultured Cells* (E005), different: *K562* (E123); (ii) source: *CD4 Naive Primary Cells* (E038), similar: *CD8 Naive Primary Cells* (E047), different: *Penis Foreskin Keratinocyte Primary Cells skin03* (E058) and *Left Ventricle* (E095). Results are reported in Table S12 and Table S13 for scenario (i) and (ii), respectively.

Table S12: Cross-cell-type prediction performance, test AUROC. Source cell-type: E003, '(s)': similar, '(d)': different epigenome.

Test cell	Intra-cell	Cross-cell (E003)	Variation (%)
E005 (s)	0.7548	0.7554	0.08
E123 (d)	0.8171	0.8118	−0.66

In the Tables we report the test AUROC performance obtained by training ShallowChrome on the training set of the test epigenome ('Intra-cell'), the test performance obtained by training on the training set of the source epigenome ('Cross-cell'), and the relative performance variation. As expected,

Table S13: Cross-cell-type prediction performance, test AUROC. Source cell-type: E038, '(s)': similar, '(d)': different epigenome.

Test cell	Intra-cell	Cross-cell (E038)	Variation (%)
E047 (s)	0.8152	0.8119	−0.41
E058 (d)	0.7446	0.7277	−2.27
E095 (d)	0.7378	0.7139	−3.24

we observe similar or mildly reduced drops in performances in similar cell types, and a more consistent decrease in different cell types. Interestingly, the model fitted on the H1 Cell Line (epigenome E003) reasonably generalises on the K562 cell line (epigenome E123), despite this last one being related to chronic myelogenous leukemia patient, thus suggesting a stable epigenome in the transformation. On the other hand, the model from CD4 naive cells (epigenome E038) has more difficulties in generalising over completely unrelated cell types, even if the relative AUROC decrease is only in the range of few per cent. Looking over these results we may hypothesise that the role of the considered HMs in predicting gene expression is rather universal, and that the small quantitative differences in their weights are those that allow to fine tune the prediction.

S8 Reproducing AttentiveChrome results

Contrary to DeepChrome [1], the AttentiveChrome [2] paper does not provide cell-type specific test results. Nonetheless, we found that the authors provided the set of pretrained models via the Kipoi model repository[1]. In order to obtain the AUROC test performance reported in Figure 2 of the main manuscript we thus downloaded each of these models and ran them in inference mode over the test set data of the respective epigenome. We note that we could not retrieve the pretrained model – and not even the dataset – for epigenome E059. After having collected all predictions for the available models, we computed test AUROC and AUPR scores via the Python Sci-kit Learn library.

Having reproduced the AttentiveChrome model predictions for 55 out of 56 epigenomes, we were also able to compute additional test performance metrics that were not reported in the original paper. In particular, we computed AttentiveChrome test AUPR scores, which we report in terms of aggregated statistics in Table S14, along with those for our ShallowChrome model in standard configuration. The results of our model do not exactly match those in Table S3 as we manually excluded the result for (the missing) epigenome E059 from the computation.

Table S14: Aggregated statistics on the test AUPR-scores for ShallowChrome and AttentiveChrome computed across 55 out of the 56 original epigenomes (E059 has been excluded from the computation).

Statistic	AttentiveChrome	ShallowChrome
Mean	0.4922	0.8469
Median	0.4768	0.8575
Max	0.8552	0.8988
Min	0.2321	0.7746

Results show that ShallowChrome dramatically outperforms AttentiveChrome according to this classification metric. Results in terms of AUPR clearly unveil the severe overfitting experienced by this deep learning model on certain specific epigenomes (see Min statistic).

References

[1] Singh, R., *et al.*: DeepChrome: Deep-learning for predicting gene expression from histone modifications. Bioinformatics **32**(17), 639–648 (2016). doi:10.1093/bioinformatics/btw427

[2] Singh, R., *et al.*: Attend and predict: Understanding gene regulation by selective attention on chromatin. Adv Neural Inf Process Syst **30**, 6785–6795 (2017). doi:10.1101/329334

[1] `https://kipoi.org/models/AttentiveChrome/`

[3] Kundaje, A., *et al.*: Integrative analysis of 111 reference human epigenomes. Nature **518**(7539), 317–330 (2015). doi:10.1038/nature14248

[4] Bannister, A., Kouzarides, T.: Regulation of chromatin by histone modifications. Cell Res **21**(3), 381–395 (2011). doi:10.1038/cr.2011.22

[5] Frasca, F., *et al.*: Exposing and characterizing subpopulations of distinctly regulated genes by k-plane regression. In: Raposo, M., Ribeiro, P., Sério, S., Staiano, A., Ciaramella, A. (eds.) Computational Intelligence Methods for Bioinformatics and Biostatistics. LNBI (Lecture Notes in Bioinformatics), vol. 11925, pp. 227–238. Springer, Heidelberg, D (2020). doi.org/10.1007/978-3-030-34585-3_20
